# Estimating the Extraction Time of an Upper Third Molar: Proposal and Validation of Results

**DOI:** 10.3390/diagnostics14182075

**Published:** 2024-09-19

**Authors:** Belén Lima-Sánchez, Paula Hermida-Cabrera, Vanessa Montoya-Salazar, Luis-Guillermo Oliveros-López, Pedro Alomar-Velasco, Maria-Angeles Serrera-Figallo, Daniel Torres-Lagares, María Baus-Domínguez

**Affiliations:** 1 Department of Stomatology, Faculty of Dentistry, University of Seville, 41009 Seville, Spain; belima97@gmail.com (B.L.-S.); paulahermidacabrera@gmail.com (P.H.-C.); vane843@hotmail.com (V.M.-S.); lgoliveros7@gmail.com (L.-G.O.-L.); danieltl@us.es (D.T.-L.); 2 Oral and Maxillofacial Unit, Quirónsalud Palmaplanas Hospital, 07010 Mallorca, Spain; doctoralomar35@hotmail.com

**Keywords:** classification, diagnostic procedure, difficulty indices, extraction, impacted third molars, validation, wisdom teeth

## Abstract

**Background:** Numerous studies in the literature have aimed to evaluate the difficulty level of removing third molars. However, most of these studies have focused on the lower third molars, which can lead to complications. There is a lack of a method to determine the complexity of upper third molar extraction. Therefore, this study’s objective was to develop an equation using multiple linear regression to estimate the extraction time of an upper third molar based on its complexity. **Methods:** This study involved patients enrolled in the Master of Oral Surgery program at the University of Seville. To determine their relationship with surgical time, the researchers analyzed various factors, such as depth, root morphology, and the need for odontosection. They then validated their findings by studying patients treated at Palmaplanas Hospital in Mallorca. **Results:** The cohort analysis from the University of Seville revealed significant associations between surgical time and the identified factors. A regression equation design was performed to predict the total duration of surgical intervention for wisdom teeth extraction. This equation incorporates several independent variables, represented by Xi, together with a constant term, C, and the corresponding coefficients, Bi, which weight the impact of each variable on the intervention time. The results are as follows: −0.312 (spatial relationship), 0.651 (depth), −0.443 (bone and mucosa integrity), 0.214 (roots), −0.745 (ostectomy), 0.713 (odontosection), and −0.426 (suture). Upon application of the statistical methodology to the Palmaplanas Hospital cohort, a regression coefficient of 0.770 was determined. This indicates a strong correlation between the input data and the estimated surgical time. **Conclusions:** In conclusion, the proposed formula demonstrates notable validity in predicting the surgical time required to extract upper third molars.

## 1. Introduction

Third molar extraction is one of the most frequent procedures in daily surgical practice, which is why the morbidity of complications can be significant [1,2]. Before starting surgery, it is essential to know the degree of difficulty of the procedure so that the best surgical approach can be designed, including all the measures aimed at minimizing the risk of complications and iatrogenic damage [1,3,4,5,6,7,8,9,10,11,12,13,14]. Likewise, knowing the degree of difficulty of the extraction will be a valuable tool for communicating with the patient and presenting from a more objective point of view the possibility of complications in the case and the expected results. It will also help the professional to decide to refer the patient if necessary. In addition, knowing the difficulty of the extraction will make it easier to determine an approximate surgical time and the appointment time with the patient [15]. In this way, it will be possible to optimize the cabinet time.

Many studies have attempted to evaluate the difficulty of extracting third molars; however, most of the literature focuses on the procedure for extracting lower wisdom teeth, with very few studies referring to the upper third molars. In most of the articles, the analyses focus on radiological evaluation. However, some authors indicate that it is challenging to know the degree of difficulty of extraction only with a preoperative radiological examination, and some warn that only intraoperatively can the degree of difficulty be known [1,3,5,6,16,17,18].

One of the most recognized evaluation techniques is the Winter classification from 1926, which categorizes the angulation of an impacted third molar in relation to the second molar’s longitudinal axis [14]. This was followed by the method of Pell and Gregory in 1933. However, the Pell and Gregory scale has been reported to be of limited value for extraction difficulty [5,7,12,16,18,19,20,21], even in vertical wisdom teeth [7,16]. Generally, classifications based solely on third molar position do not adequately predict difficulty [7,22]. However, classifications that include clinical and radiological features have recently begun to be proposed to achieve standardized assessment knowledge [23].

Pederson (1988) proposed the joint angulation analysis and Pell and Gregory classification [8,19,24]. This method has been compared with other indexes numerous times, and all authors share the same opinion about it. The Pederson index has a deficient predictive value and is, therefore, not helpful in evaluating the surgical difficulty of the mandibular third molar [2,3,6,7,8,9,10,12,16,17,18,20,25]. It should be noted that the Pederson index is designed only with radiographic variables and that variables such as bone density, the shape and size of the roots, and their relationship with the inferior alveolar nerve (IAN), among others, are not evaluated [3,16,26].

Years earlier, in 1976, MacGregor developed the first semiquantitative model relating extraction time to surgical difficulty, called the WHARFE index (Winter classification, height of the mandible, angulation of the second molar, root shape and morphology, follicle development, path of exit of the tooth during removal) [20]. The fact that it is an index exclusively based on radiographic characteristics and its complexity makes it a minimally used index [16]. However, compared to Pederson’s index, the WHARFE evaluation is more consistent and of more excellent reliability [17].

The surgeon must rely on the available scientific evidence to estimate the degree of surgical difficulty prior to the extraction of a wisdom tooth. However, most published articles proposing indexes to calculate the difficulty of third molar extraction focus on the extraction of the lower third molars, based almost exclusively on radiographic variables and their relationship to the lower dental nerve [27], which can lead to a more significant number of complications in upper third molar surgeries due to the lack of a method that allows us to know the complexity of such extractions.

The main objective of this study was to develop and validate a predictive model of the difficulty of upper cord extraction using specific clinical and radiographic variables. We sought to validate this model with an independent dataset to verify its robustness and applicability in different clinical contexts.

## 2. Materials and Methods

### 2.1. Study Design

This study presents the same methodology as was applied in our previously published manuscript on the lower third molars [28]. Although it presents the same study design, the present manuscript works with a different sample of wisdom teeth, in this case, superior, and the results obtained are not at all the same as those reported in the previous study.

This research was carried out as a prospective cohort study, receiving approval from the Research Ethics Committee of the University of Seville under protocol code 1336-N-23. This study aligns with the ethical guidelines outlined in the Declaration of Helsinki for medical research involving human subjects.

The participants or their guardians were thoroughly informed of the purpose of this study and provided written informed consent for both their participation and the surgical procedure.

The only invasive intervention carried out was the extraction of third molars that had an absolute indication for removal.

### 2.2. Patient Enrollment

This study included patients who were attended by second- and third-year students of the Master’s Degree in Oral Surgery of the University of Seville, Faculty of Dentistry, diagnosed with third-molar dysodontiasis, and underwent surgery in the Master’s Degree in Oral Surgery and Advanced Oral Surgery.

All patients included in the study had to meet specific criteria for participation:

- Diagnosis of third molar dysodontiasis.
- Requirement for extraction of the third molar due to absolute indications (presence of infection episodes, presence of caries impossible to restore or caries in the adjacent second molar impossible to restore without removing the wisdom tooth, presence of periodontal lesion due to the position of the third molar and its relationship with the second molar, cases of dentigerous cyst or other related pathology, cases of external resorption of the third molar or the second molar due to the presence of the wisdom tooth).
- Availability of a comprehensive medical history.
- Had undergone a radiological examination (orthopantomography).
- Provided informed consent after thorough explanation.

The exclusion criteria for the study included the following:

- The presence of severe or uncontrolled medical conditions, such as cardiovascular diseases, diabetes, blood coagulation disorders, or any other condition that might pose risks during oral surgery.
- High risk of causing injury to neighboring anatomical structures, notably the maxillary sinus.
- History of surgical complications from previous third molar extractions, such as bone fractures or severe postoperative infections.

Each patient underwent an individual assessment to evaluate these exclusion criteria. This evaluation considered the patient’s complete medical and dental records, along with any pertinent factors related to the extraction of the upper third molar. Relevant data were then collected post-procedure using a structured data collection form (Table 1). The time of the intervention was recorded from the moment the incision was made, except for those cases in which the teeth erupted, where the intervention was scheduled from the moment of the syndemostomy.

### 2.3. Surgical Intervention

The extractions of upper wisdom teeth involved the use of local anesthesia consisting of 4% articaine combined with 1:100,000 epinephrine (Ultracaín, Normon, Madrid, Spain).

In the case of partially or fully impacted wisdom teeth, a carefully designed scalloped or bayonet mucoperiosteal flap ensured optimal access to the surgical site.

Ostectomy procedures were performed using a number 8 tungsten carbide round bur and handpiece, with continuous irrigation for optimal visibility and cooling. The odontosection was performed using a Lindemann drill and turbine.

The luxation and subsequent extraction of the wisdom teeth were performed using straight extractors of varying widths, supplemented by winter extractors as deemed necessary.

Upon extraction, a thorough irrigation of the alveolus was performed to ensure the removal of any residual bone or tooth fragments, with particular attention given to the removal of the follicular sac.

The distal aspect of the second molars in contact with the surgical area was curetted using Gracey Curettes 13/14 to promote proper periodontal integration in the operated region. Suturing was performed using Mayo needle holders and non-absorbable suture material Supramid Aragó 4/0 or 5/0 TB12—CT 16mm 3/8.

The patients received comprehensive written and verbal postoperative instructions and were scheduled for a follow-up appointment after one week for suture removal and evaluation.

A standard pharmacological regimen was prescribed consisting of 400–600 mg of ibuprofen every 8 h for 5–7 days in conjunction with paracetamol 650–1000 mg every 8 h for 5–7 days. In cases where surgery extended beyond 2 h, required significant ostectomy, or involved patients with active infections or compromised immune systems, a 500 mg amoxicillin prescription was provided every 8 h for 3 days, with a review on the third day to determine the need to continue the antibiotic regimen, adhering to the guidelines outlined in the National Antibiotic Resistance Plan and the Antimicrobial Therapeutic Guide of the Spanish National Health System.

### 2.4. Data Analysis

The information about the upper jaw surgery was recorded on specially designed cards to capture various extraction details. Subsequently, all data were organized into a table using SPSS 9.0 (IBM, Armonk, NY, USA) for statistical analysis.

A comprehensive descriptive examination was conducted, providing a thorough overview of all variables.

To estimate the duration of the procedure in minutes, a multiple linear regression approach using the backward method was used. This involved analyzing the values of different continuous or discrete variables.

Statistical significance levels were denoted in standard format (*p* < 0.05; *p* < 0.01; *p* < 0.001, *p* < 0.0001, and *p* < 0.00001), where lower values indicate greater significance.

### 2.5. Validation

During the validation phase of the study, the subjects were individuals diagnosed with third molar dysodontiasis at Hospital QuirónSalud Palmaplanas in Palma de Mallorca, Spain. The procedures were performed by a single experienced surgeon.

A comprehensive descriptive analysis provided detailed information on all variables under consideration. To assess the associations between qualitative variables, the Chi test^2^ was employed. Identifying specific groups exhibiting significant differences was accomplished using Haberman’s corrected standardized residuals. This statistical approach allowed us to determine if the frequency of observations within a particular cell significantly deviated from the expected value based on the total sample size, thus indicating statistical significance.

The Mann–Whitney U test was employed to assess the association between numerical variables due to their non-normal distribution.

Spearman’s correlation coefficient was utilized to examine the correlations between variables.

## 3. Results

### 3.1. Individuals and Profile

This study involved 46 patients from the University of Seville, who were used to calculate coefficients for multiple linear regression. Furthermore, 72 patients from Palmaplanas Hospital in Mallorca were included to validate the results obtained from the University of Seville sample (Table 2).

### 3.2. Variables Associated with the Upper Third Molars Included in the Sample

The table below (Table 3) displays the data pertaining to the variables linked to the upper wisdom teeth that underwent surgery in the sample.

### 3.3. Regression Equation

The regression equation used in this study was devised to forecast the total duration of surgical intervention for wisdom tooth extraction. This equation incorporates several independent variables, represented by Xi, along with a constant term, C, and the corresponding coefficients, Bi, which weigh the impact of each variable on the intervention time. The formula is presented as:

Y = C + B_1_ × X_1_ + B_2_ × X_2_ + B_3_ × X_3_ +…

Y represents the total duration of the intervention. C denotes the model’s constant (found in column B under the constant row in Table 4). Bi represents the value in column B for each variable in the model, while Xi signifies the value of the independent variable corresponding to B.

Table 4 provides a summary of the coefficients attributed to each variable, calculated following the application of the regression equation following surgeries performed on the patient cohort of the University of Seville.

**Figure 1:**
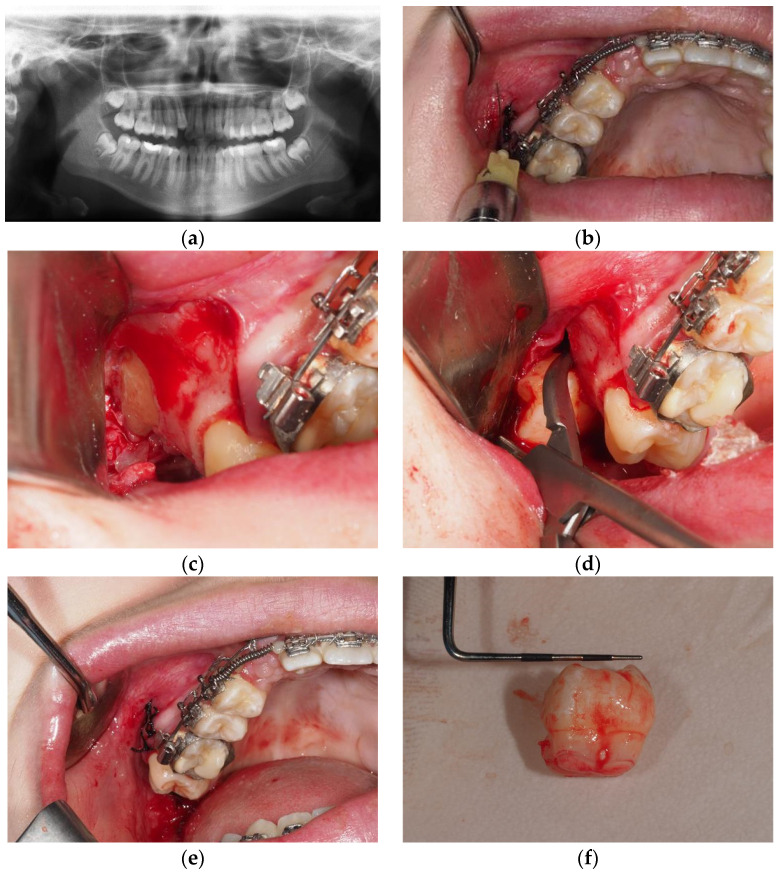
Extraction of an upper level C deep wisdom tooth vertical, covered with mucosa but not bone with less than 2/3 of fused root without the need for osteotomy or odontosection and suture; Y = C + B1 × X1 + B2 × X2 + B3 × X3 + B4 × X4 + B5 × X5 + B6 × X6 + B7 × X7= 9.442 − 0.312 × 1 + 0.651 × 3 − 0.443 × 4 + 0.214 × 1 − 0.745 × 1 + 0.713 × 1 − 0.426 × 2 = 8.641 min of extraction. (**a**) Orthopantomography; (**b**) anesthesia; (**c**) full thickness flap; (**d**) exodontia without osteotomy or odontosection; (**e**) suture; (**f**) exo-donated third molar germ.

Xi, representing the value of the independent variable for each Bi, is assigned a value ranging from 1 to 5 based on its difficulty index (derived from previously published indexes). A value of 1 indicates the least challenging scenario, while 3, 4, or 5 (depending on the variable) signifies the most complex conditions for lower third molar extraction (refer to Table 5) (Figure 1).

The table below displays the statistical values of the prognosis and the absolute difference between the actual extraction time of the third molar and the estimated time calculated using the regression equation (Table 6).

The mean difference between the actual extraction time and the estimated time, calculated using the regression equation, is zero. This is a deliberate feature of the model, as the regression line is constructed to minimize the average discrepancy between the observed and predicted values.

### 3.4. Validation Results

To validate the equation, an evaluation was carried out on a dataset comprising 72 patients treated at Palmaplanas Hospital in Mallorca. The regression equation, initially formulated using data from the University of Seville (as described in Table 4), was applied to these cases. To address occasional negative estimates generated by the equation for procedures classified as the simplest, a correction was implemented to ensure a minimum performance duration of two minutes. This adjustment aimed to improve the practical applicability of the model and maintain its accuracy in various surgical scenarios (refer to Table 7).

To estimate the Mallorca data, we used the regression equation derived from the Seville dataset (as depicted in Table 3). To mitigate the occasional negative estimates resulting from procedures clas-sified as the simplest, a correction was applied to ensure a minimum performance duration of two minutes. This adjustment was intended to enhance the accuracy and practical utility of the estima-tion process.

In conclusion, a table presenting a summary of the model has been provided for reference (Table 8).

Upon examination, it is evident that upon applying the coefficients derived from the patient data obtained in Seville to those in Mallorca, a correlation coefficient (r) of 0.807 was obtained. This signifies a notably strong positive correlation, approaching the highest possible value of 1.

To facilitate comprehension of the statistical analysis, it is essential to note that the regression equation was utilized, yielding a series of coefficients for each variable (refer to Table 4). The constants (B) were calibrated using a computer algorithm to optimize coefficients for all variables. Consequently, only statistically significant variables with a *p*-value < 0.001 were retained. These crucial variables, as depicted in both Table 4 and Table 5, encompass aspects such as the spatial orientation of the upper wisdom tooth (vertical, mesioangular, distoangular, or horizontal/angular), depth (classified as A, B, or C according to Pell–Gregory), integrity of the bone and mucosa (ranging from fully erupted to entirely covered by bone and mucosa), root morphology (including fusion and multiplicity), performance of odontosection and ostectomy procedures, and the necessity of suturing.

To ensure the robustness of the findings and to prevent overreliance on internal validity alone (derived from the Seville cohort of 46 patients), the results were validated using a separate sample of 72 patients from Mallorca. Applying the multiple linear regression equation yielded a correlation coefficient (r) of 0.807, which aligns closely with the maximum possible value of 1.

## 4. Discussion

Estimating the surgical difficulty of wisdom tooth extraction remains a subject of debate because of the subjectivity to which the procedure may be exposed, especially about the operator.

In the first place, one might think that a lack of clinical experience on the part of the surgeon would limit the ability to predict the surgical difficulty of the procedure. Some authors, such as Komerik et al., observed no differences in determining the difficulty level of third molar extraction between surgeons with different experience levels [5]. However, others, such as Ferrus-Torres, demonstrated in their publication that when trying to determine the difficulty level of third molar extraction, surgeons with less experience made more errors in this estimation [5]. Similarly, Sanchez-Jorge et al., 2023 [22] concluded that dental training significantly influenced the perceived difficulty of third molar extraction since dentists without surgical training saw the intervention as more difficult on visual analog scales. In contrast, professionals with postgraduate training in oral surgery considered patient-related factors of greater relevance than operative factors. In the study by Komerik et al., 2014 [5], even though senior surgeons and residents took the same amount of time at each difficulty level and no statistically significant differences were observed, they did observe that as the complexity of the surgical act increased, the ability to correctly predict extraction difficulty was more equivocal, with residents more commonly overestimating the difficulty of the procedure, while experienced surgeons underestimated more cases. These results conclude that preoperative assessment of the difficulty level of third molar extraction is unreliable despite more excellent surgical experience. This is in line with the statement of some authors [1,3,6] that the true complexity of the procedure is only known intraoperatively. The study by Komerik et al., 2014 [5] evaluated the surgical difficulty based on clinical factors such as age, sex, patient weight, and access to the surgical site (oral cavity opening and tongue interference), along with radiographic factors using the Pell and Gregory classification along with the Winter classification, the periodontal and dental follicle space, the shape, number, and degree of root formation, and its proximity to the inferior dental nerve. However, the level of difficulty was estimated using an “intuitive calculation” after considering the sum of the clinical and radiographic factors mentioned above. The fact that scales based solely on the position of the wisdom tooth, such as Pell and Gregory’s and Winter’s, do not adequately predict the level of difficulty [5,7,12,13,16,19,20,21,29], together with the fact that the type of calculation performed in the study by Komerik et al., 2014 [5], may lead us to think that although there are differences in deciding the level of difficulty of extraction between experienced and novice surgeons, perhaps part of this difference is in the method used for prediction. Nevertheless, and given that no differences were observed in the study regarding the surgical time between one group and the other, it is proposed that two years is sufficient to achieve skill in performing third molar extraction without complications, something that is of interest given that, in our study, all the extractions were carried out by second and third-year students of the Master’s Degree in Oral Surgery of the University of Seville.

Jaron et al., 2021 [29] also used several classifications in their study, including Winter and Pell and Gregory, to determine the degree of impaction and to determine a better methodology for the surgical procedure, relating them to the age of the patient and other factors, such as the bone matrix around the impacted third molar, degree of ankylosis, and the weight of the patient. Their results revealed that the procedures were significantly easier in patients under 20 years of age, with the degree of difficulty increasing up to 30 years of age, having as a limitation that it is only based on the location of the impacted tooth. The rest of the factors mentioned also influence clinical judgment, but in this case, they are based on the operator’s experience. Gay-Escoda et al., 2022 [13] carried out a systematic review in which they compiled the existing scales to evaluate the difficulty of third molar extraction, observing that they are mainly based on radiographic variables and very few consider the surgical variables or the experience of the operator, thus emphasizing the importance of a protocol that includes all the variables to facilitate treatment planning, help prevent possible complications and if necessary refer the patient to a specialist with proven experience.

Regarding the duration of surgery, Bhuju et al. (2018) [30] published an article in which they observed the relationships between the patient’s age, sex, side of impaction, and type of impaction and difficulty. It was observed that the most statistically significant factors were sex and impaction; in contrast, age and impact type showed no significant correlation. According to modified difficulty indexes, Ku et al., 2020 [31] showed that operative time was highly correlated with the patient’s pathological conditions. On the other hand, Komerik et al., 2014 [5] related operative time to extraction difficulty, indicating that the extractions that were more complex correlated with more extended time. However, on some occasions, the time required to complete the operation did not depend solely on the variables previously studied. Still, almost half of the causes for the longer time required were due to other factors that were not predicted before starting, such as the patient’s ability to keep the mouth open during the procedure. As in the study above, Osunde et al., 2015 [24] evaluated the effects of age, sex, and level of surgical difficulty according to the Pederson criteria; the results showed that these variables did not affect pain or the limitation of opening after third molar surgery. This is interesting because, at first, our study focused on whether there was a relationship between the different items studied and surgical time (Table 3 and Table 4). No statistically significant results were obtained, except for the time of ostectomy. However, our study did not consider patient-dependent variables, such as the ability to adequately open the mouth and cheek flexibility.

An article published by Lambade et al., 2023 [18] proposed a new index to evaluate the difficulty of extraction, the Lambade–Dawane–Mali (LDM) preoperative index. It is intended to measure the association between the preoperative and the LDM difficulty score index with three different variables: postoperative surgical maneuvers using the modified Parant scale (MPS), perioperative time, and the gold standard, the Pederson index. This new MLD index uses operative time as the primary variable and demographic (age and gender) and clinical variables as covariates. It differs from other traditional indexes by considering these variables together with the radiological ones, making it superior due to its precision, simplicity, reproducibility, and highly significant results using the concordance between methods and the Kappa statistic. Therefore, the authors conclude that preoperative assessment helps anticipate difficulty, plan the approach, and improve time scheduling in clinical practice. Bhansali et al., 2021 [16] reviewed the literature, including all the relevant indices proposed to date, where, like other authors mentioned [1,18], they attached importance to the need for an index that can be used to predict preoperative difficulty. However, they considered an index that provides precious information of greater importance to avoid erroneous judgments. In their article, Carvalho et al., 2018 [8] concluded that more studies are needed to evaluate the types of complications that may arise during surgery and correlate them with preoperative factors.

Other studies, such as that by Roy et al., 2015 [6], where they compared the Pederson index with a new index, stated that the time of surgery is the gold standard to pre-decide the difficulty level of an extraction. They show a Kappa of agreement of 89.0% when relating their proposed new index and the time spent in the extraction versus the Kappa of agreement value of 66.50% of the Pederson index, highlighting that there is no agreement between the Pederson index and the time of the operation. The new index proposed by this group includes the evaluation of factors such as depth from the point of elevation, opening of the patient’s oral cavity, size of the tongue, angulation of the external oblique ridge, flexibility of the cheeks, and width and curvature of the roots, which again emphasizes that the Pederson index is insufficient for the prediction of the surgical difficulty of a third molar [3,6,7,8,9,10,12,16,17,20,25,29]. Similarly, Sekhar et al., 2021 [17] compared the Pederson and WHARFE indices with WAR lines, concluding that the WHARFE evaluation is more consistent and reliable than the Pederson index, as are the studies mentioned above since WHARFE takes into account the root morphology as an additional factor not included in the Pederson index. However, the study by Roy et al., 2015 [6] reported that there is indeed a correlation between the difficulty determined preoperatively by its index and the time it takes to complete the operation. Still, it does not predict the surgical time before surgery.

Despite the promising findings of this study, it is important to recognize certain limitations that can affect the interpretation and generalization of the results. The cohorts of patients in Seville and Mallorca presented significant differences in several key variables. These differences can influence the generalization of the regression model derived from the Seville data when applied to the population of Mallorca. Although the model showed a strong correlation coefficient (R = 0.807) and a reasonable ability to predict extraction times in Mallorca, these differences could potentially introduce biases that were not fully controlled.

Furthermore, clinical practices may vary between different centers and professionals, which could affect the applicability of the model in other contexts. The frequency of procedures such as odontosection and suturing differs between the centers studied, suggesting that the results may not be directly extrapolated to other clinical settings without additional adjustments. To mitigate the negative predictions generated by the regression equation in procedures classified as the simplest, a correction was applied to ensure a minimum duration of two minutes. Although this adjustment improves the practical applicability of the model, it may not accurately reflect all possible clinical scenarios and suggests the need for a more dynamic approach in future research.

The sample size used to derive the model in Seville consisted of 46 patients, while the validation was carried out with 72 patients from Mallorca. While this provides a reasonable basis for validation, a larger sample size and greater diversity in patient characteristics could further strengthen the robustness of the model and its ability to generalize to broader populations.

To address these limitations, future studies are recommended to consider including patient samples from multiple centers with diverse clinical practices to validate and fit the model, perform additional analyses to evaluate the influence of demographic and clinical differences on outcomes, and develop more robust and dynamic models that can adapt to variations in clinical practices and patient characteristics.

Another significant limitation is the exclusion of explicit consideration for complications during the extraction of impacted wisdom teeth within the multiple linear regression model. While several relevant variables, such as depth, spatial position, relationship with the second molar, need for osteotomy, odontosection, and root morphology were meticulously evaluated, the potential occurrence of intraoperative complications, such as excessive bleeding or unforeseen issues, was not explicitly addressed. These unexpected complications can profoundly impact procedural duration, underscoring the importance of future research endeavors incorporating measures to assess and mitigate such complications. By doing so, predictive models’ precision and clinical applicability for extraction time could be enhanced.

Furthermore, another limitation lies in the variability of operator experience during the extraction of impacted wisdom teeth. While one group of cases was managed by students under supervision as part of their Master of Oral Surgery program at the University of Seville, another group was handled by a seasoned oral surgeon. This discrepancy in operator experience could have influenced various facets of the procedure, including technical proficiency, clinical decision making, and the management of potential complications, potentially impacting the study outcomes.

Moreover, this study did not account for certain patient-derived factors, such as facial morphology’s influence on procedural complexity or ethnic characteristics affecting surgical intervention. Addressing these factors in future research could provide a more comprehensive understanding of the complexities involved in impacted wisdom tooth extraction.

Considering these limitations, it may be beneficial to conduct validation studies of the regression model across different academic institutions and participant demographics to ascertain the clinical significance and generalizability of the proposed formula.

## 5. Conclusions

This study focused on the development and validation of a predictive model for the difficulty of upper cord extraction, with the main objective of improving surgical planning in dentistry. A multiple linear regression equation was formulated that showed a significant proportion (R = 0.807), using specific clinical and radiographic variables to predict the extraction time. This advancement represents an objective tool that can facilitate better communication with patients about possible complications and expected results.

However, this study acknowledges certain limitations that could affect the generalizability of the results. There were significant differences between the groups of patients in the Seville and Mallorca centers that could influence the applicability of the model in different clinical contexts. Furthermore, variations in clinical practices and operator experience between different centers suggest the need for additional adjustments to improve model accuracy in different settings.

Comparison of the model’s performance with traditional extraction difficulty indices was positive, highlighting its potential to outperform existing methods in terms of the accuracy and reliability. Key clinical and radiographic variables that affect removal difficulty are identified, providing a solid foundation for future research and improvements in clinical practice.

Moving forward, it is recommended to expand the sample size and diversify the characteristics of the patients to strengthen the robustness of the model. Furthermore, incorporating explicit assessment of intraoperative complications and considering operator experience could further improve the clinical applicability of the model. In summary, although this study represents a significant step toward more accurate surgical planning, future research and validation in multiple clinical settings is essential to maximize the utility and generalizability of the proposed predictive model.

## Figures and Tables

**Table 1:** Intervention data sheet.

Intervention Time	Minutes
Identification	Left Right
Spatial relationship	Mesioangular Horizontal/transverse Vertical Distoangular
Depth	Level A Level B Level C
Branch/2m distal face ratio	Class I Class II Class III
Bone and mucosal integrity	Partially covered by mucosa Partially covered by bone and mucosa Covered only by mucosa, not by bone Covered by mucosa and partially by bone Entirely covered by mucosa and bone
Roots	More than 2/3 merged More than 2/3 separated or less than 1/3 separated More than 2/3, multiple
Follicle size	>1 mm 0 mm
Actions (more than one box can be checked)	Osteotomy Odontosection Sutura Simple exodontia

**Table 2:** Variables in different centers.

Variables	Categories	Seville		Mallorca		Sign. *
		Frequency	Percentage	Frequency	Percentage	
Sex	Man	19	38.9	27	41.3	
	Woman	27	61.1	44	59.7	
Age (categorized)	Up to 22 years old	7	29.3	22	15.9	<0.01
	From 23 to 29 years old	21	26.7	20	47.71	
	From 30 to 39 years old	12	16.0	12	27.3	
	40 or more years	4	28.0	21	9.11	
Ostectomy	Yes	16	25.3	19	34.8	
	No	30	74.7	56	65.2	
Odontosection	Yes	9	4.0	3	19.62	<0.01
	No	37	96.0	72	80.42	
Suture	Yes	27	38.7	29	58.71	<0.05
	No	19	61.3	46	41.31	
Simple exodontia	Yes	34	74.7	56	73.9	
	No	12	25.3	19	26.1	

* Significant differences: Values were given only for those differences that were statistically significant.

**Table 3:** Variables associated with the upper third molars included in the sample.

Variables	Categories	Seville		Mallorca		Sign.*
		Frequency	Percentage	Frequency	Percentage	
Spatial relationship	Mesioangular	3	6.5	7	9.3	
	Horizontal/angled	0	0.0	0	0.0	
	Vertical	39	84.8	42	56.0	<0.01
	Distoangular	4	8.7	26	34.7	
Depth	Level A	23	50.0	52	69.3	quasi
	Level B	17	37.0	16	21.3	
	Level C	6	13.0	7	9.3	
Bone and mucosal integrity	Partial mucosa	34	81.0	51	75.0	
	Partial bone and mucosa	1	2.4	0	0.0	
	Total mucosa, no bone	6	14.3	6	8.8	
	Total mucosa and bone	1	2.4	11	16.2	
Roots	>2/3 merged	30	65.2	67	89.3	<0.001
	>2/3 separated or <1/3 separated	15	32.6	5	6.7	
	>2/3 multiple	1	2.2	3	4.0	
Difficulty index (categorized)	Not very difficult	36	78.3	53	70.7	
	Difficult	9	19.6	19	25.3	
	Very difficult	1	2.2	3	4.0	
Difficulty index (dichotomous)	Not very difficult	36	78.3	53	70.7	
	Difficult or very difficult	10	21.7	22	29.3	

* Significant differences: Values were given only for those differences that were statistically significant.

**Table 4:** Coefficients for each variable from the cases operated at the University of Seville.

Variable	Non-Standardized Coefficients		Coef. Est.	t	Sig (p)
	B	Error	Beta		
(Constant)	9.442	6.446		1.465	0.151
Spatial relationship	−6.780	2.704	−0.312	−2.507	0.017
Depth	11.031	2.736	0.651	4.032	0.000
Bone and mucosal integrity	−4.665	1.919	−0.443	−2.431	0.020
Roots	4.846	2.238	0.214	2.166	0.037
Ostectomy	−18.610	4.483	−0.745	−4.152	0.000
Odontosection	21.401	5.616	0.713	3.811	0.000
Suture	−10.298	3.434	−0.426	2.999	0.005

**Table 5:** Value of Xi for each variable as a function of extraction difficulty.

Variable	Categories	Value of Xi
Spatial relationship	Vertical	1
	Mesioangular	2
	Distoangular	3
	Horizontal/angled	4
Depth	Level A	1
	Level B	2
	Level C	3
Bone and mucosal integrity	Erupted	1
	Partial mucosa	2
	Partial bone and mucosa.	3
	Total mucosa, no bone	4
	Total mucosa and bone	5
Roots	>2/3 merged	1
	>2/3 or <1/3 separated	2
	>2/3 multiple	3
Ostectomy	No	1
	Yes	2
Odontosection	No	1
	Yes	2
Suture	No	1
	Yes	2
Simple exodontia	Yes	1
	No	2

**Table 6:** Statistical metrics of the forecast and the absolute variance between the actual and estimated time.

Forecast		Forecast Error (Actual Value—Estimate)				
Media	Standard Deviation	Media	Standard Deviation	Q1	Q2	Q3
11.52	9.71	0.00	7.11	−3.96	−1.29	3.29

**Table 7:** The statistical metrics of the forecast and the absolute variance between the actual and estimated times were applied to the dataset from Mallorca.

Forecast		Forecast Error (Actual Value—Estimate)				
Media	Standard Deviation	Media	Standard Deviation	Q1	Q2	Q3
6.35	6.93	1.62	6.55	−2.11	3.00	4.00

**Table 8:** Overview of the model.

R	R Square	Adjusted R-Squared	Standard Error of the Estimate
0.807	0.651	0.587	7.733

## Data Availability

The authors will make the raw data supporting this article’s conclusions available upon request.
